# Influence of Heat Treatment Condition on the Microstructure, Microhardness and Corrosion Resistance of Ag-Sn-In-Ni-Te Alloy Wire

**DOI:** 10.3390/ma17112785

**Published:** 2024-06-06

**Authors:** Ling Shao, Shunle Zhang, Liepeng Hu, Yincheng Wu, Yingqi Huang, Ping Le, Sheng Dai, Weiwei Li, Na Xue, Feilong Xu, Liu Zhu

**Affiliations:** 1 Zhejiang Provincial Key Laboratory for Cutting Tools, Taizhou University, Taizhou 318000, China; shaoling@tzc.edu.cn (L.S.); newpalemoon@outlook.com (Y.W.); yingqihuang77@hotmail.com (Y.H.); sdai@tzc.edu.cn (S.D.); lww@tzc.edu.cn (W.L.); a731634915@hotmail.com (F.X.); 2 Zhejiang Key Laboratory for Island Green Energy and New Materials, Taizhou University, Taizhou 318000, China; 3 Ningbo Electric Alloy Material Co., Ltd., Ningbo 315040, China; zhangshunle@nbeaf.com (S.Z.); hulp@nbeaf.com (L.H.); yuep@nbeaf.com (P.L.)

**Keywords:** Ag-Sn-In-Ni-Te alloy, heat treatment, microstructure, microhardness, corrosion resistance

## Abstract

Ag-Sn-In-Ni-Te alloy ingots were produced through a heating–cooling combined mold continuous casting technique; they were then drawn into wires. However, during the drawing process, the alloy wires tended to harden, making further diameter reduction challenging. To overcome this, heat treatment was necessary to soften the previously drawn wires. The study investigated how variations in heat treatment temperature and holding time affected the microstructure, microhardness and corrosion resistance of the alloy wires. The results indicate that the alloy wires subjected to heat treatment at 700 °C for 2 h not only exhibited a uniform microstructure distribution, but also demonstrated low microhardness and excellent corrosion resistance.

## 1. Introduction

Ag-based electrical contact materials such as AgMeO [1], AgNi [2], AgC [3], AgWC [4] and AgZrB_2_ [5] are presently among the most widely employed options in the field. Various AgMeO electrical contact materials, including AgCdO [6], AgSnO_2_ [7], AgZnO [8], AgZnO_2_ [9], AgCuO [10], AgY_2_O_3_ [11] and Ag(MgCoNiCuZn)O [12] have been developed due to their exceptional electrical contact properties, improved resistance to welding and arc erosion, as well as enhanced thermal and electrical conductivity. While AgCdO electrical contact material exhibits remarkable electrical contact properties, its utilization is increasingly limited because of the toxicity of Cd during both production and usage. Nonetheless, the release of hazardous Cd vapor during operation can lead to severe health issues in humans. The AgSnO_2_ electrical contact material, known for its environmental friendliness, is widely seen as the perfect alternative to AgCdO in electrical contact applications. Compared with AgCdO contact material, the AgSnO_2_ contact material possesses superior welding resistance. AgSnO_2_ has emerged as a leading choice for medium- and low-voltage electrical contact materials currently, attracting significant attention from numerous research teams actively investigating its properties.

The properties of electrical contact materials, such as processability and switching behavior, are heavily influenced by the fabrication method employed [13]. It is widely acknowledged that refining the preparation method can boost the performance of AgSnO_2_ electrical contact materials. Several investigations indicate that the key characteristics of materials, such as their processability and switching behavior, are closely tied to their microstructure; this, in turn, is significantly influenced by the fabrication method employed. There are diverse techniques available for the preparation of Ag-based electrical contact materials, including internal oxidation [14], reactive ball milling [15], powder metallurgy-spark plasma sintering [16], chemical coating-sintering-extrusion [13], cold isostatic pressing-sintering-hot extrusion [17] and so on. In contrast to other conventional approaches, AgSnO_2_ materials produced through internal oxidation demonstrate reduced contact resistance and less temperature elevation [13]. However, alloy ingots are prerequisites for internal oxidation to take place. The heating–cooling combined mold (HCCM) continuous casting represents an innovative processing method for acquiring superior ingots characterized by exceptional deformability and surface quality [18]. This is attributed to the substantial temperature gradient observed at the leading edge of solid–liquid interfaces in the casting direction. Consequently, the utilization of HCCM continuous casting yields refined casting microstructures and a more even dispersion of alloying elements, surpassing outcomes achievable through traditional casting methods [19].

The AgSnO_2_ electrical contact material experiences notable challenges, including significant contact resistance, elevated temperature increases and inadequate stability during prolonged service [20]. This is attributed to the aggregation of SnO_2_ particles on the contact surface over extended periods of use; this is a consequence of the limited wettability between SnO_2_ and Ag. During the past decade, various nonmetals, metals or metallic oxides—including N [21], F [22], Cr [7], Ni [21], Cu [23], Y [24], Bi [25], La [26], Ce [27], Gd [28], CuO [29], Cu_2_O [30], NiO [31], ZnO [32], TiO_2_ [33], WO_3_ [34], Bi_2_O_3_ [33], In_2_O_3_ [35] and La_2_O_3_ [36]—have been employed as additives to enhance the anti-arc erosion behavior, mechanical properties and conductivity of AgSnO_2_ contact material. Among these additives, the addition of In_2_O_3_ demonstrated remarkable enhancements in the ultimate strength and elasticity moduli of AgSnO_2_ contact materials, along with improvements in cyclic creep behavior. In the automotive sector, AgSnO_2_In_2_O_3_ contacts have demonstrated favorable performance in high-voltage switchgear devices. Optimizing the addition of Ni enhanced the temperature resistance and expanded the lifespan of AgSnO_2_In_2_O_3_ contacts [37]. Adding Te or rare earth metals to Ag-Sn-In alloys helps suppress the formation of surface oxidation layers and they act as nucleation sites for the formation and growth of Sn and In oxides, resulting in finer microstructures [38]. The inclusion of Te in Ag-Sn-In alloy significantly enhances its performance during the later stages of the internal oxidation process [39]. Compared with AgSnO_2_In_2_O_3_ without additives, the additive CuO has little effect on electrical durability, while the additive TeO_2_ can significantly improve the electrical life [40].

In this study, Ag-Sn-In-Ni-Te alloy ingots were fabricated using the HCCM continuous casting technique, and subsequently drawn into wires. During the drawing process, Ag-Sn-In-Ni-Te alloy wires tended to harden, hindering further diameter reduction. To facilitate further diameter reduction, the previously drawn Ag-Sn-In-Ni-Te alloy wires required heat treatment for softening. The impact of heat treatment temperature and holding time on the microhardness and corrosion resistance of Ag-Sn-In-Ni-Te alloy wires were investigated, in preparation for subsequent internal oxidation.

## 2. Experimental

Ag-Sn-In-Ni-Te alloy was prepared by utilizing high-purity metals Ag (99.99%, GB/T 4135-2016), Sn (99.99%, GB/T 728-1998), In (99.993%, YS/T 257-2009), Ni (99.99%, GB/T 26016-2021) and Te (99.99%, YS/T 222-2010) as the raw materials. The theoretical values of chemical composition of Ag-Sn-In-Ni-Te alloy are given in Table 1. The melting process was conducted in a customized high-frequency induction melting furnace. Initially, 53.7 kg of Ag blocks were placed into the furnace and melted into liquid Ag at a temperature of 1050 °C. Subsequently, 3.56 kg of Sn blocks, 1.48 kg of In blocks, 0.09 kg of Ni blocks and 0.47 kg of Te blocks were sequentially added and melted at 1050 °C. Once all pure metal blocks were completely melted, the molten mixture was stirred with a graphite rod to ensure uniform mixing of the five metals. Throughout the melting process, a 15 mm thick layer of charcoal (with individual charcoal dimensions of 20 mm × 15 mm × 15 mm) was placed above the metallic ingots to isolate the molten metal from contact with air, thus preventing oxidation.

**Figure 1:**
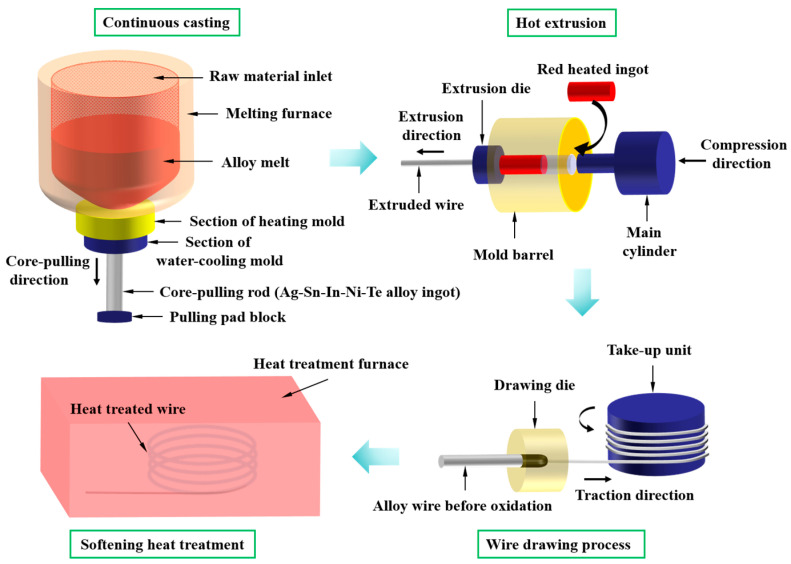
Schematic diagram of Ag-Sn-In-Ni-Te alloy wire preparation process.

For HCCM continuous casting, a pulling pad block with a withdrawal device was utilized (as shown in Figure 1). The withdrawal device was moved downwards at a velocity of 200 mm min^−1^, with a pause of 2 s every 8 mm of movement. Simultaneously, a cooling water switch was opened, and the automatic withdrawal device was activated. Liquid metal in the wedge-shaped groove of the pulling pad block as the core-pulling rod (Ag-Sn-In-Ni-Te alloy ingot) moved downwards. Cooling and solidification were facilitated by positioning Cu sleeves filled with circulating cooling water around the outer surface of the water-cooling mold. Upon withdrawing the ingot from the water-cooling mold, a secondary water-cooling process was initiated when the gap between the bottom of the ingot and the bottom of the water-cooling mold exceeded 100 mm. The flow rate of the cooling water was maintained at 7 m^3^ h^−1^. Following the HCCM continuous casting process, a cylindrical ingot measuring 90 mm in diameter and 1000 mm in height was produced. The chemical composition of the cylindrical ingot measured by an EDX-LE energy dispersive X-ray fluorescence spectrometer (EDX, SHIMADZU, Kyoto, Japan) is shown in Table 1. The measured values of chemical composition of Ag-Sn-In-Ni-Te alloy are relatively close to the theoretical values. Afterward, the cylindrical Ag-Sn-In-Ni-Te alloy ingot underwent heating in a protective atmosphere of decomposed ammonia, to 720 °C, followed by hot extrusion into wires with a diameter of 4.8 mm. Subsequently, the Ag-Sn-In-Ni-Te alloy wire underwent successive drawing processes, reducing its diameter to 4.3 mm and then to 3.9 mm; ultimately achieving a final wire diameter of 3.9 mm.

Divide the 3.9 mm diameter Ag-Sn-In-Ni-Te alloy wire into six groups, assigning one group as the control group, while subjecting the remaining five groups to annealing treatment in a tube furnace atmosphere with decomposed ammonia. Table 2 outlines the specifics of the heat treatment conditions. For clarity, the samples undergoing different heat treatment conditions were denoted as 700—2 h, 750—2 h, 800—2 h, 800—5 h and 850—5 h, respectively. To examine the microstructures of both the cross-section and longitudinal section of the Ag-Sn-In-Ni-Te alloy wire, samples were wire-cut into cylindrical shapes with dimensions of Φ3.9 mm × 10 mm for cross-section observation, and half-cylinders with the same dimensions for longitudinal section observation. Subsequently, both the cross-sections and longitudinal sections underwent wet grinding with abrasive papers, followed by mechanical polishing to attain mirror-smooth surfaces, and were then cleaned in alcohol using ultrasonic agitation. Etchant solution comprised 100 mL NH_3_·H_2_O and 100 mL H_2_O_2_ was utilized to etch the cross-sections and longitudinal sections of the specimens for examination of morphology and microstructure. Morphology and microstructure were characterized using optical microscopy (OM, Zeiss AxioScope A1, Carl Zeiss, Jena, Thuringia, Germany) and field-emission scanning electron microscopy (SEM, Hitachi S-4800, Hitachi, Tokyo, Japan). Crystal phase analysis was performed using X-ray diffraction (XRD, Bruker D8 Advance, Bruker Corp., Billerica, MA, USA) employing Cu Kα radiation within a 2*θ* range of 10° to 90° at room temperature. The step size and rate were set at 0.02° and 3° per minute, respectively.

Microhardness evaluations were carried out on both the samples before and after heat treatment utilizing an HMV-G micro-Vickers hardness tester (Shimadzu Co, Ltd., Kyoto, Japan) on the polished longitudinal section at room temperature. A load of 100 gf was applied for a dwell time of 10 s. Each specimen underwent testing a minimum of ten times at random positions to derive an average value and standard deviation, indicative of the specimen’s microhardness. To avoid additional stresses from interactions between consecutive indentations, a standard spacing between indentations (at least three times the diagonal length) was maintained.

Electrochemical tests were conducted in a 3.5 wt.% NaCl aqueous solution exposed to air at 25 °C utilizing a CS350M electrochemical workstation (Wuhan Corrtest Instruments Corp., Ltd., Wuhan, China). A standard three-electrode configuration was utilized within a 180 mL glass cell. This setup included an exposed rectangular sample serving as the working electrode, a platinum plate as the counter electrode, and saturated calomel electrode (SCE) acting as the reference electrode. The cylindrical samples were mounted vertically in silica gel, exposing their cross-sections (approximately 0.1 cm^2^). Sample preparation involved successive grinding with SiC papers up to 5000 grit, followed by a concluding polish using 0.25 μm diamond paste. Afterward, the samples were rinsed in alcohol and dried with a stream of air. Each test was conducted on a freshly prepared electrode surface, with all potentials measured relative to an SCE. The open circuit potential (OCP) was monitored for 1 h before experiments commenced, ensuring its stability across all alloys. Subsequently, potentiodynamic polarization tests were carried out by scanning the specimens at a speed of 0.33 mV s^−1^ within the range of −500 mV to 800 mV relative to the OCP. More than three electrochemical tests were carried out for Ag-Sn-In-Ni-Te alloy samples before and after heat treatment, enabling the determination of mean values and standard errors of the mean.

## 3. Results and Discussion

### 3.1. Structural, Morphological and Chemical Characterization

**Figure 2:**
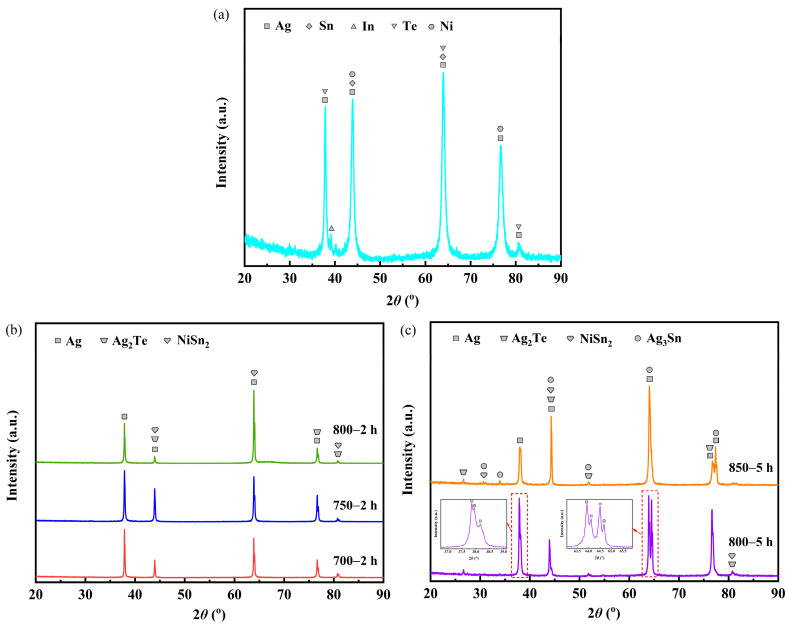
XRD patterns for the samples before and after heat treatment: (**a**) sample untreated; (**b**) samples 700—2 h, 750—2 h and 800—2 h; (**c**) samples 800—5 h and 850—5 h.

Figure 2 presents the XRD patterns for the samples before and after heat treatment. The sample labeled as untreated before heat treatment consisted of phases of Ag (PDF-#04-0783), Sn (PDF-#04-0673), In (PDF-#05-0642), Te (PDF-#27-0871) and Ni (PDF-#04-0850) (Figure 2a). Analysis of the XRD patterns (Figure 2b) indicates that samples 700—2 h, 750—2 h and 800—2 h comprised phases of Ag, Ag_2_Te (PDF-#42-1266 and PDF-#45-1399) and NiSn_2_ (PDF-#08-0430). Furthermore, samples 800—5 h and 850—5 h consisted of phases of Ag, Ag_2_Te, NiSn_2_ and Ag_3_Sn (PDF-#01-071-0530) (Figure 2c).

**Figure 3:**
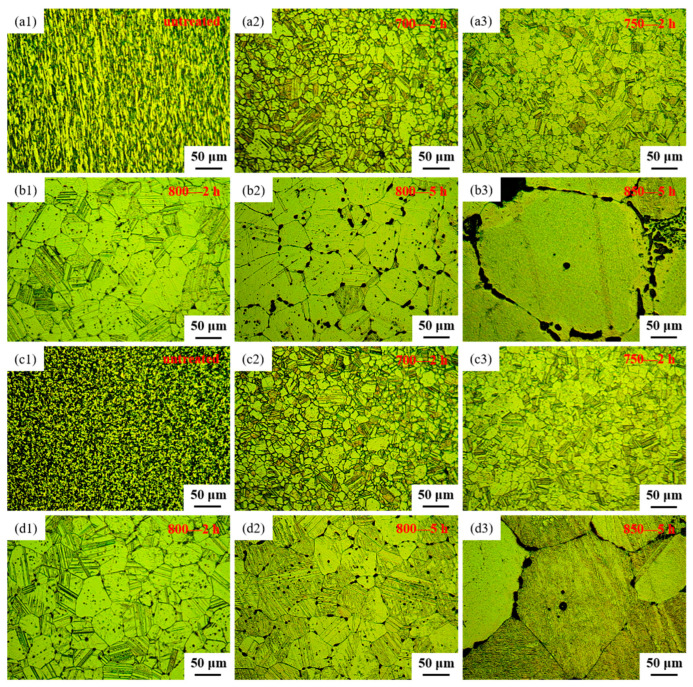
Optical microstructure of Ag-Sn-In-Ni-Te alloy samples before and after heat treatment: (**a1**) longitudinal section of sample untreated, (**a2**) longitudinal section of sample 700—2 h, (**a3**) longitudinal section of sample 750—2 h, (**b1**) longitudinal section of sample 800—2 h, (**b2**) longitudinal section of sample 800—5 h, (**b3**) longitudinal section of sample 850—5 h, (**c1**) cross-section of sample untreated, (**c2**) cross-section of sample 700—2 h, (**c3**) cross-section of sample 750—2 h, (**d1**) cross-section of sample 800—2 h, (**d2**) cross-section of sample 800—5 h, (**d3**) cross-section of sample 850—5 h.

The microstructural features of both the longitudinal sections and cross-sections of samples before and after heat treatment, observed through OM, are depicted in Figure 3. Figure 3(a1) and Figure 3(c1), respectively, represent the longitudinal and cross-sectional views of the sample untreated. From Figure 3(a1,c1), it can be observed that the alloy, after extrusion and wire drawing, exhibits fine grains with a uniform distribution of black phases. These black phases appear as a linear distribution in the longitudinal section and as uniformly dispersed dots in the cross-section, indicating that the black phase existed in a fibrous form within the alloy. Figure 3(a2,c2) depict the microstructure of sample 700—2 h, in longitudinal and cross-sectional views, respectively. Comparing the microstructure of the longitudinal section between sample untreated and sample 700—2 h, it becomes evident that the linear black phase transforms into black dots after heat treatment. Moreover, the microstructure of the cross-section of sample 700—2 h tends to align with that of the longitudinal section. Upon contrasting the microstructure of the cross-section between sample untreated and sample 700—2 h, the grain boundaries of sample 700—2 h become more distinct, the black phase decreases, and the grain size increases. Figure 3(a3,c3) display the microstructure of sample 750—2 h in longitudinal and cross-sectional views, respectively. Upon comparing the microstructure between sample 700—2 h and sample 750—2 h, it was observed that by increasing the heat treatment temperature to 750 °C, the grain size increased, and black dots were distributed both at grain boundaries and within grains. Additionally, clear twin structures are noticeable in samples 700—2 h, 750—2 h, 800—2 h, 800—5 h and 850—5 h (Figure 3). The creep strength of Ag-based alloys can be significantly improved by the development of twin structures [41]. From Figure 3, it is apparent that the percentage of grain-boundary area in various samples of Ag-Sn-In-Ni-Te alloy follows the sequence: sample 700—2 h > sample 750—2 h > sample 800—2 h > sample 800—5 h > sample 850—5 h. An increased proportion of grain-boundary area is anticipated to promote accelerated oxygen diffusion, thus expediting the internal oxidation process [42].

Figure 3(b1,d1) present the microstructure of sample 800—2 h in longitudinal section and cross-section, respectively. A comparison of the microstructure between sample 750—2 h and sample 800—2 h reveals that, as the heat treatment temperature increased to 800 °C with a constant holding time, the grain size nearly doubled, and the amount and size of the black phase increased. Figure 3(b2,d2) illustrate the microstructure of sample 800—5 h in the longitudinal section and cross-section, respectively. Comparing the microstructure between sample 800—2 h and sample 800—5 h, it can be observed that with a constant heat treatment temperature and extended holding time, the grain size continued to increase, and the amount and size of the black phase increased further. Additionally, the precipitation of the black phase at grain boundaries became notably more pronounced. Furthermore, with the prolongation of holding time, the black phase gradually changed its morphology from circular dots to ellipses, short rods, L-shapes, C-shapes and so on. The microstructures of sample 850—5 h are illustrated in Figure 3(b3,d3) for longitudinal and transverse views, respectively. From Figure 3(b3,d3), it can be seen that there was a considerable enlargement in the grains of the alloy wire after heat treatment at 850 °C for 5 h. The presence of the black phase within the grains noticeably diminished, mainly relocating along the grain boundaries. The black phase adhered closely to the contours of the grain boundaries, presenting either a continuous or partially continuous arc-shaped distribution, or forming dense dot-like clusters along these boundaries.

**Figure 4:**
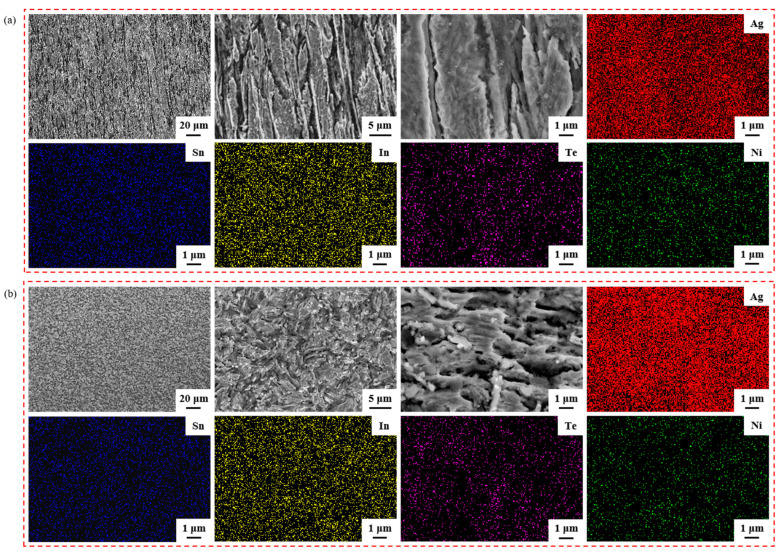
Element mapping results for the longitudinal section and cross-section of sample untreated. Group (**a**) images are SEM micrographs and EDS spectra showing the distribution of Ag, Sn, In, Te and Ni elements in the longitudinal section; group (**b**) images are SEM micrographs and EDS spectra showing the distribution of Ag, Sn, In, Te and Ni elements in the cross-section.

Figure 4 illustrates the element mapping results for both the longitudinal section and the cross-section of the sample untreated. Group Figure 4a images are SEM micrographs and EDS spectra showing the distribution of Ag, Sn, In, Te and Ni elements in the longitudinal section; group Figure 4b images are SEM micrographs and EDS spectra showing the distribution of Ag, Sn, In, Te and Ni elements in the cross-section. The second and third images depict SEM micrographs at a higher magnification of the first image in group Figure 4a,b. From the EDS spectra, it is evident that the elements Ag, Sn, In, Te and Ni were evenly dispersed within the sample untreated.

**Figure 5:**
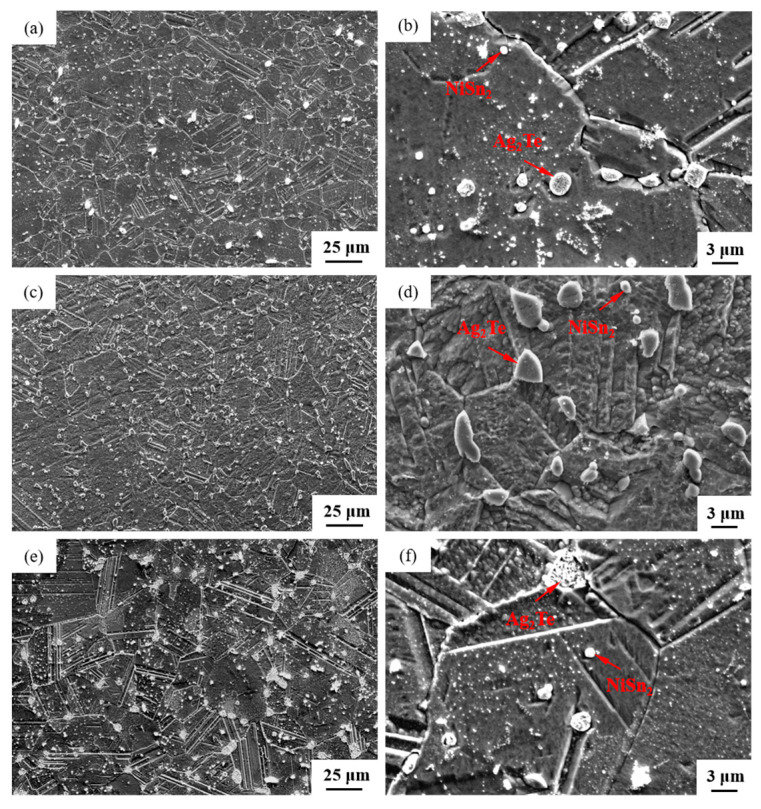
SEM microstructure of Ag-Sn-In-Ni-Te alloy samples after different heat treatments: cross-section of sample 700—2 h (**a**,**b**), cross-section of sample 750—2 h (**c**,**d**) and cross-section of sample 800—2 h (**e**,**f**).

**Figure 6:**
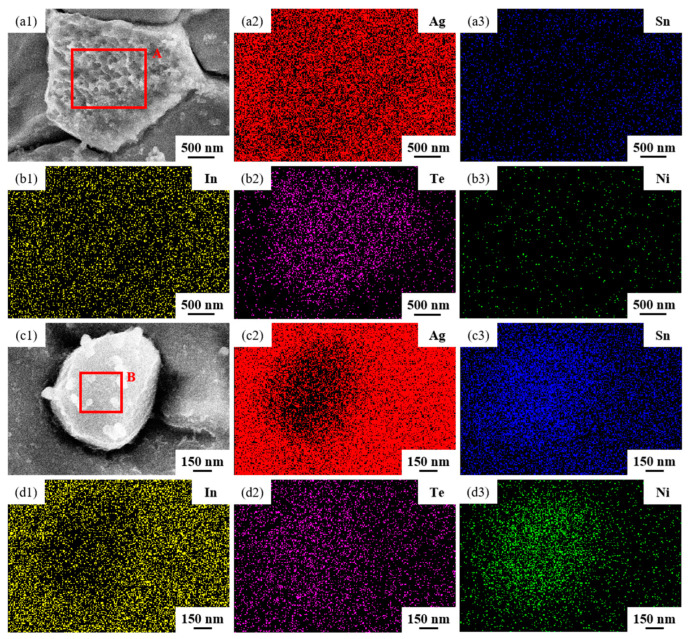
Element mapping results for two distinct size particles distributed across matrix of sample 700—2 h. SEM micrographs (**a1**,**c1**); the distribution of Ag (**a2**,**c2**), Sn (**a3**,**c3**), In (**b1**,**d1**), Te (**b2**,**d2**) and Ni (**b3**,**d3**) elements.

**Figure 7:**
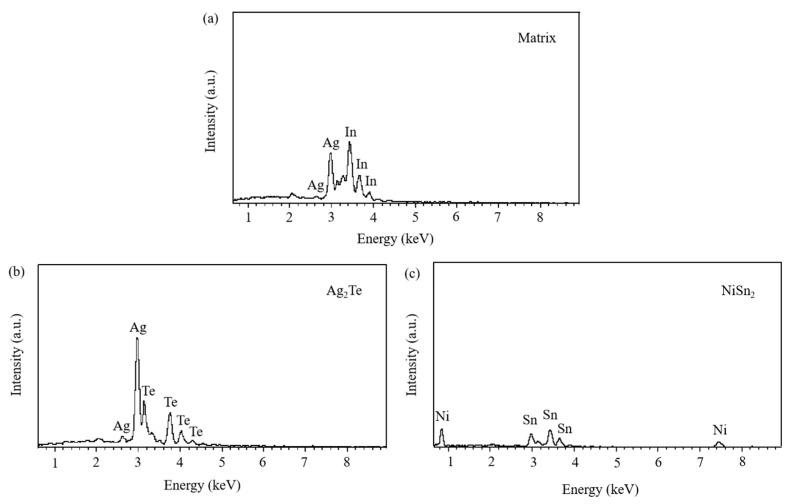
Spectra results of matrix and two distinct size particles distributed across matrix of sample 700—2 h. Matrix (**a**); Ag_2_Te (**b**), NiSn_2_ (**c**).

The SEM micrographs in Figure 5 depict the microstructures of samples 700—2 h, 750—2 h and 800—2 h of the Ag-Sn-In-Ni-Te alloy after undergoing heat treatments at 700 °C, 750 °C and 800 °C for 2 h, respectively. From Figure 5, it is evident that samples 700—2 h, 750—2 h and 800—2 h each display two distinct size particles distributed across their respective matrices. Element mapping analysis was conducted for these differing size particles in Figure 6(a1) and Figure 6(c1), respectively. Figure 6(a2,a3,b1,b3) illustrate the distribution of Ag, Sn, In, Te and Ni elements of Figure 6(a1), respectively. Notably, the large particle in Figure 6(a1) exhibits a prominent presence of Ag and Te elements. Similarly, Figure 6(c2,c3,d1–d3) depict the distribution of Ag, Sn, In, Te and Ni elements of Figure 6(c1), respectively, with the small particle predominantly containing Sn and Ni elements. The spectra results in Figure 7 illustrate the composition of the matrix and two distinct size particles distributed within the sample 700—2 h. From Figure 7, it can be inferred that the matrix consists of an Ag-In solid solution, while the large particles are composed of Ag-Te intermetallic, and the small particles are composed of Ni-Sn intermetallic. To delve deeper into the composition of the large particle and the small particle, area scanning analysis was performed in regions A and B of Figure 6(a1,c1), respectively, yielding the results outlined in Table 3. The area scanning analysis reveals that the composition of region A (large particle) comprises 71.29 at.% Ag and 28.71 at.% Te, while region B (small particle) consists of 34.07 at.% Ni and 65.93 at.% Sn. This suggests that the phase of region A corresponds to Ag_2_Te phase, while region B corresponds to NiSn_2_ phase. Comparing Figure 5a,c,e, it appears that as the heat treatment temperature increased from 700 °C to 800 °C, not only did the grain sizes gradually increase in samples 700—2 h, 750—2 h and 800—2 h, but the size of the Ag_2_Te and NiSn_2_ phases also gradually increased. Additionally, more Ag_2_Te phase aggregated at the grain boundaries. When the grain size within the wire enlarged and brittle Ag_2_Te intermetallic compounds aggregated at the grain boundaries, the material’s workability became extremely poor. If the additive Te aggregated at the grain boundaries in the form of Ag_2_Te, when using such an alloy for internal oxidation to prepare AgSnO_2_In_2_O_3_ materials, the final performance may not meet expectations due to the uneven distribution of TeO_2_ phase.

**Figure 8:**
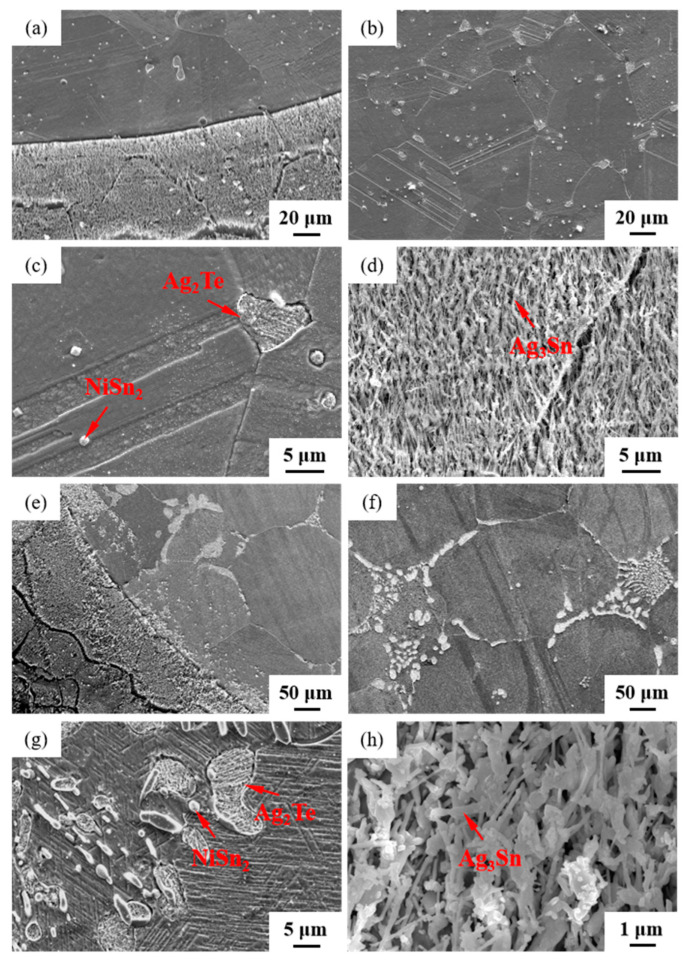
SEM microstructure of Ag-Sn-In-Ni-Te alloy samples after different heat treatments: cross-section of sample 800—5 h (**a**–**d**); cross-section of sample 850—5 h (**e**–**h**).

**Figure 9:**
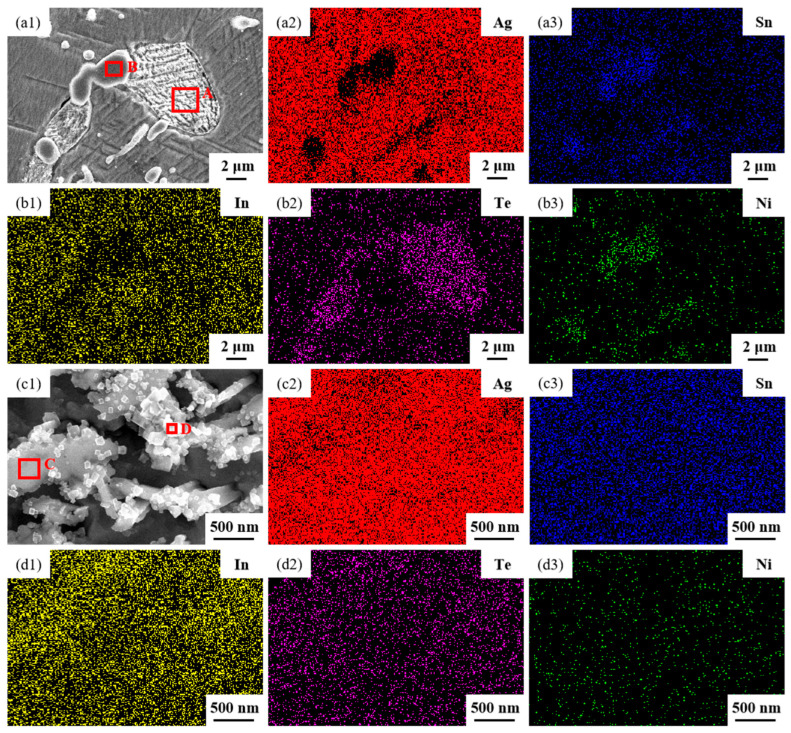
Element mapping results for different phases distributed across a matrix of sample 850—5 h. SEM micrographs (**a1**,**c1**); the distribution of Ag (**a2**,**c2**), Sn (**a3**,**c3**), In (**b1**,**d1**), Te (**b2**,**d2**) and Ni (**b3**,**d3**) elements.

The microstructures of samples 800—5 h and 850—5 h of the Ag-Sn-In-Ni-Te alloy, observed via SEM, are presented in Figure 8 after undergoing distinct heat treatments at 800 °C and 850 °C for 5 h, respectively. From Figure 8a,e, it appears that when the heat treatment temperature surpassed 800 °C and the duration extended beyond 5 h, filamentous substances emerged at the edges of samples 800—5 h and 850—5 h. Figure 8a–c depict SEM images progressively magnified; similarly, Figure 8e–g also display SEM images progressively magnified. Samples 800—5 h and 850—5 h exhibit two particles of different sizes and shapes distributed across the matrix’s middle position. One type of particle appeared relatively large with irregular shapes, while the other type was small and elliptical. Element mapping analysis was performed for these particles in Figure 9(a1). Figure 9(a2,a3,b1–b3) illustrate the distribution of Ag, Sn, In, Te and Ni elements in Figure 9(a1), respectively. Particularly noteworthy is that the large particle in Figure 9(a1) exhibits a significant presence of Ag and Te elements, whereas the small particle predominantly contains Ni and Sn elements. To delve deeper into the composition of the large and small particles, area scanning analysis was carried out in regions A and B of Figure 9(a1), yielding the results summarized in Table 4. The area scanning analysis reveals that the composition of region A (large particle) comprises 66.68 at.% Ag and 33.32 at.% Te, while region B (small particle) consists of 33.76 at.% Ni and 66.24 at.% Sn. This suggests that the phase of region A corresponds to Ag_2_Te, while region B corresponds to NiSn_2_. Figure 8d,h present magnified SEM images of the filamentous substances at the edges of samples 800—5 h and 850—5 h, respectively. Element mapping analysis was conducted for these filamentous substances in Figure 9(c1). Figure 9(c2,c3,d1–d3) illustrate the distribution of Ag, Sn, In, Te and Ni elements of Figure 9(c1), respectively. However, the elements do not exhibit a clear tendency towards aggregation. To further investigate the composition of the filamentous substances, area scanning analysis was performed in regions C and D of Figure 9(c1), yielding the results outlined in Table 4. The area scanning analysis reveals that the composition of region C comprises 69.70 at.% Ag and 30.30 at.% Sn, and region D consists of 75.59 at.% Ag and 24.41 at.% Sn. This indicates that both regions C and D correspond to the phase Ag_3_Sn. The elements Te, Sn and Ni in the Ag-Sn-In-Ni-Te alloy did not dissolve uniformly in the crystal of Ag as In did, but mainly existed in the form of intermetallic compounds Ag_2_Te and NiSn_2_ within the Ag-Sn-In-Ni-Te alloy. Upon heating to 300 °C–600 °C, Ag_2_Te phase partially decomposed, and the decomposed Ag_2_Te reaggregated and recombined. After the heat treatment temperature exceed 800 °C, the reaggregation and recombination effects of the Ag_2_Te phase became most pronounced. When the heat treatment temperature surpassed 850 °C (below the alloy’s melting point) and was held for a sufficient duration, the alloy grain boundaries were converged by the Ag_2_Te phase. This is because at high temperatures, the degree of reaggregation and recombination of the Ag_2_Te phase was greater, and at grain boundaries, due to the presence of interfacial energy, interfacial energy reshaped the recombined the Ag_2_Te phase to match the contour of the grain boundaries.

### 3.2. Microhardness

**Figure 10:**
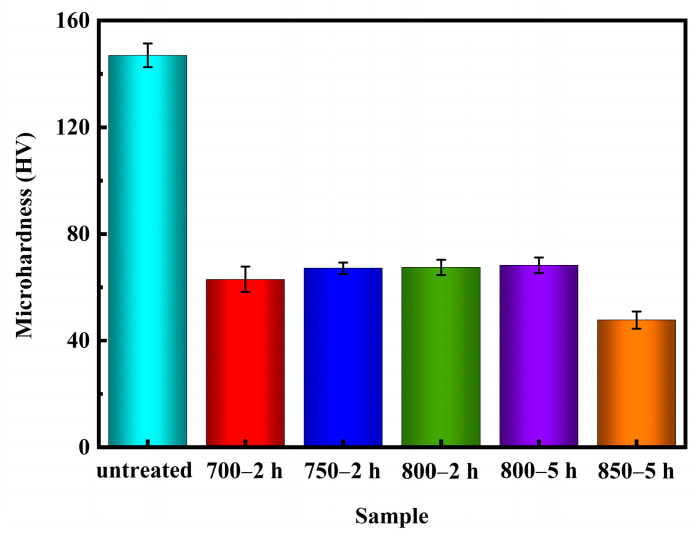
Microhardness of Ag-Sn-In-Ni-Te alloy samples before and after heat treatment.

The Vickers hardness tests were performed following the guidelines outlined in the ASTM E384-17 standard [43] test method. Figure 10 illustrates the microhardness values of Ag-Sn-In-Ni-Te alloy samples before and after various heat treatments. The data plotted on the graph (Figure 10) is an average value of ten measurements. It is evident from Figure 10 that the microhardness values of the samples decreased significantly after heat treatment. Specifically, the microhardness value of sample 700—2 h was lower compared to samples 750—2 h, 800—2 h and 800—5 h. However, the microhardness value of sample 700—2 h was higher than the microhardness value of sample 850—5 h. Despite sample 850—5 h exhibiting the lowest microhardness value, filamentous substances were observed at its edges, resulting in a non-homogeneous microstructure compared to samples 700—2 h, 750—2 h and 800—2 h.

### 3.3. Potentiondynamic Polarization Curves

**Figure 11:**
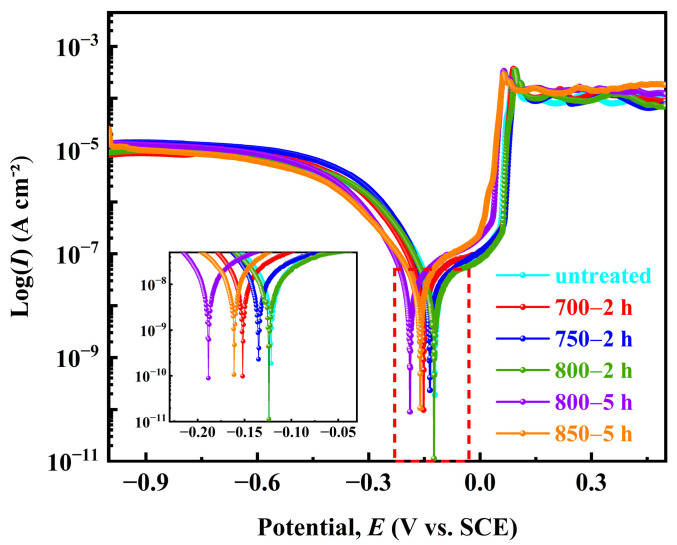
Potentiondynamic polarization curves of the prepared samples in 3.5% NaCl solution at room temperature.

The polarization curves of the five Ag-Sn-In-Ni-Te alloy samples in the 3.5% NaCl solution are shown in Figure 11. It can be seen that, from the shape of the polarization curves, the overall polarization curves of the samples follow the sequence from left to right: sample 800—5 h → sample 850—5 h → sample 700—2 h → sample 750—2 h → sample 800—2 h → sample untreated. The corrosion potentional (*E*_corr_) and corrosion current density (*I*_corr_) of the five samples were calculated using the Tafel extrapolation method, as shown in Table 5. The corrosion tendencies of the five Ag-Sn-In-Ni-Te alloy samples were similar, and their corrosion potentials were similar. The *E*_corr_ from low to high was as follows: sample 800—5 h < sample 700—2 h < sample untreated < sample 800—2 h < sample 750—2 h < sample 850—5 h. The *I*_corr_ from high to low was as follows: sample 850—5 h > sample 800—5 h > sample 800—2 h > sample 750—2 h > sample 700—2 h > sample untreated. The results show that the *E*_corr_ of sample 700—2 h is more negative, and the corrosion tendency is larger, but the *I*_corr_ is smaller, only 60.75 ± 6.4 cm^−2^, comparing with samples 750—2 h and 800—2 h. The Stern–Geary equation was used to calculate polarization resistance (*R*_p_) [44,45]:

(1) $$R_{p}=\frac{b_{a}b_{c}}{2.303I_{\mathrm{corr}}(b_{c}+b_{a})}$$

where *b*_a_ and *b*_c_ are anodic and cathodic Tafel slopes, respectively. The corrosion rate (*v*_corr_ in mm per year) was determined by the following equation [46]:

(2) $$\upsilon_{\mathrm{corr}}=0.00327\frac{I_{\mathrm{corr}}M}{nd}$$

where *M*, *n* and *d* are molar mass, charge number and the density of tested metal, respectively. The values *R*_p_ and *v*_corr_ calculated using Equations (1) and (2) are also shown in Table 5. The *R*_p_ was in the order: sample 850—5 h < sample 800—5 h < sample 800—2 h < sample 750—2 h < sample 700—2 h < sample untreated. Conversely, the *v*_corr_ was in the order: sample 850—5 h > sample 800—5 h > sample 800—2 h > sample 750—2 h > sample 700—2 h > sample untreated. The results show that the *R*_p_ of sample 700—2 h is larger, but the *v*_corr_ is smaller, only 1.62 mm year^−1^, compared with samples 750—2 h and 800—2 h.

### 3.4. Electrochemical Impedance Spectroscopy

**Figure 12:**
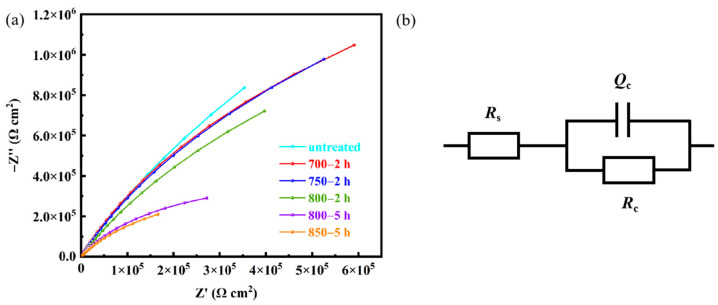
Electrochemical impedance spectroscopy of the prepared samples in 3.5% NaCl solution at room temperature (**a**) and R(CR) equivalent circuit model (**b**).

Figure 12a shows Nyquist plots of of Ag-Sn-In-Ni-Te alloy samples before and after heat treatment. In order to better analyze the Nyquist curve, the R(CR) equivalent circuit model in Figure 12b is adopted to fit the results. *R*_s_ denotes solution resistance, *R*_c_ represents the charge transfer resistance and surface corrosion product resistance and *Q*_c_ stands or the solid–liquid interface electric double layer capacitance. In terms of charge transfer resistance, the maximum *R*_c_ value appeared on sample untreated and the minimum *R*_c_ value appeared on sample 850—5 h. The *R*_c_ of sample 700—2 h is larger, comparing with samples 750—2 h and 800—2 h.

## 4. Conclusions

(1) Samples 700—2 h, 750—2 h and 800—2 h comprised phases of Ag, Ag_2_Te and NiSn_2_. With the increase in heat treatment temperature from 700 °C to 800 °C, not only did the grain sizes gradually increase in samples 700—2 h, 750—2 h and 800—2 h, but the size of the Ag_2_Te and NiSn_2_ phases also gradually increased. Additionally, more Ag_2_Te aggregated at the grain boundaries.

(2) Samples 800—5 h and 850—5 h consisted of phases of Ag, Ag_2_Te, NiSn_2_ and Ag_3_Sn. When the heat treatment temperature surpassed 800 °C and the duration extended beyond 5 h, filamentous Ag_3_Sn phases emerged at the edges of samples 800—5 h and 850—5 h, and the alloy grain boundaries were converged by the Ag_2_Te phase.

(3) In samples 700—2 h, 750—2 h, 800—2 h, 800—5 h and 850—5 h, samples 700—2 h, 750—2 h and 800—2 h exhibited a uniform microstructure distribution. Among them, sample 700—2 h demonstrated low microhardness and excellent corrosion resistance. Therefore, for Ag-Sn-In-Ni-Te alloy wires, the optimal heat treatment process was at 700 °C for 2 h.

## Figures and Tables

**Table 1:** Chemical composition of the prepared material (wt.%).

Element	Ag	Sn	In	Te	Ni
Theoretical value	90.55	6.0	2.5	0.8	0.15
Measured value	90.53	6.18	2.39	0.72	0.18

**Table 2:** Heat treatment conditions of the prepared specimens.

Sample	Untreated	700—2 h	750—2 h	800—2 h	800—5 h	850—5 h
Temperature (°C)	–	700	750	800	800	850
Time (h)	–	2	2	2	5	5

**Table 3:** Compositions of different phases in the sample 700—2 h.

Sample	A		B	
	Ag	Te	Ni	Sn
wt.%	67.74	32.26	32.84	67.16
at.%	71.29	28.71	34.07	65.93

**Table 4:** Compositions of different phases in the sample 850—5 h.

Sample	A		B		C		D	
	Ag	Te	Ni	Sn	Ag	Sn	Ag	Sn
wt.%	62.85	37.15	34.06	65.94	67.64	32.36	73.78	26.22
at.%	66.68	33.32	33.76	66.24	69.70	30.30	75.59	24.41

**Table 5:** Potentiondynamic polarization results of the prepared samples.

Sample	*E*_corr_ (mV)	*I*_corr_ (nA cm^−2^)	*b*_a_ (mV dec^−1^)	*b*_c_ (mV dec^−1^)	*R*_p_ (kΩ cm^2^)	*v*_corr_ (mm year^−1^)	*R*_s_ (Ω cm^2^)	*R*_c_ (Ω cm^2^)
untreated	−170.51 ± 24.8	42.64 ± 9.9	270.42	115.31	823.21	1.14	36.41	7679.9
700—2 h	−182.49 ± 15.5	60.75 ± 6.4	519.61	117.61	685.48	1.62	27.66	5246.95
750—2 h	−159.12 ± 24.2	66.05 ± 3.2	328.89	113.5	554.72	1.76	31.01	4860.7
800—2 h	−166 ± 21.0	80.42 ± 1.8	670.17	118.41	543.34	2.14	28.83	4248.8
800—5 h	−189.46 ± 2.0	87.55 ± 7.8	401.81	133.48	496.93	2.33	28.92	2865.9
850—5 h	−156.92 ± 4.5	91.71 ± 3.7	332.26	115.52	405.84	2.44	27.66	911.550

## Data Availability

Data are contained within the article.
